# Antibiotic exposure in pregnancy and risk of coeliac disease in offspring: a cohort study

**DOI:** 10.1186/1471-230X-14-75

**Published:** 2014-04-14

**Authors:** Karl Mårild, Johnny Ludvigsson, Yolanda Sanz, Jonas F Ludvigsson

**Affiliations:** 1 Dept. Medical Epidemiology and Biostatistics, Karolinska Institutet, Stockholm, Sweden; 2 Astrid Lindgren Children’s Hospital, Karolinska University Hospital, Solna, Sweden; 3 Div. of Paediatrics, Department of Clinical and Experimental Medicine, Linköping University, and Östergötland County Council, Linköping, Sweden; 4 Microbial Ecology and Nutrition Research Group, Institute of Agrochemistry and Food Technology, National Research Council (IATA-CSIC), Valencia, Spain; 5 Department of Paediatrics, Örebro University Hospital, Örebro, Sweden

**Keywords:** Antibiotics, Celiac disease, Microbiota, Pregnancy

## Abstract

**Background:**

The infant microbiota may play a pathogenic role in coeliac disease (CD). Antibiotic treatment in pregnancy is common and could significantly impact the infant microbiota. In this study, we aimed to investigate the association between antibiotic exposure during pregnancy and CD in offspring.

**Methods:**

Prospective questionnaire data on antibiotic exposure in pregnancy were available in 8729 children participating in the All Babies in Southeast Sweden (ABIS) cohort study, and of these 46 developed CD until December 2006. Cox regression estimated hazard ratios (HRs) for CD in the offspring among mothers exposed to antibiotics during pregnancy, with adjustment for parent-reported diary data on breastfeeding, age at gluten introduction and number of infections in the child’s first year of life.

**Results:**

Of the 1836 children exposed to antibiotics during pregnancy, 12 (0.7%) children developed CD as compared with 34/6893 (0.5%) unexposed children (HR = 1.33; 95% CI = 0.69-2.56). Risk estimates remained unchanged after adjustment for breastfeeding, age at gluten introduction and infection load in the child’s first year of life (HR = 1.28; 95% CI = 0.66-2.48).

**Conclusions:**

We found no statistically significant association between antibiotic exposure during pregnancy and CD in offspring. This lack of association may either be true or the result of limited statistical power.

## Background

Coeliac disease (CD) is a life-long autoimmune disease occurring in 1-2% of children and adults living in Western countries [1,2]. In CD, genetically predisposed individuals develop small-intestinal villous atrophy in response to dietary gluten intake [3]. In the last decades the prevalence of CD has more than doubled, [4] suggesting that environmental factors other than gluten-exposure may have a significant influence on CD development [5]. Earlier research has in particular emphasized the importance of environmental factors early in life, including pregnancy and the perinatal period, [6,7] for the development of CD.

The microbial colonization of the infant’s gut is considered to be critical for the appropriate development of the intestinal immune system, the establishment of oral tolerance and the mucosal barrier function [8]. Likewise, imbalances in the intestinal microbiota (dysbiosis) are frequently related to immune dysregulation and development of immune-mediated diseases and vice versa. Several studies have found dysbiosis in individuals with CD as compared with healthy controls, suggesting that the gut microbiota may play a pathogenic role in CD [9]. The infant gut colonization begins perinatally and is strongly influenced by the maternal microbiota, the mode of delivery and subsequently by the infant feeding practice [10]. Some data also indicate that maternal antibiotic intake in pregnancy influences the gut microbiota in the offspring, [11] and that perturbations caused by antibiotics in the infant [12] and adult microbiota [13,14] may persist for several years. Antibiotics are in fact one of the main environmental stressors that lead to the replacement of symbiotic bacteria by otherwise under-represented potentially pathogenic bacteria [13,15].

Today, some 10-25% [16,17] of pregnant women use antibiotics. Recent data have shown a positive association between antibiotic use during pregnancy and offspring asthma, [18] a disease that shares potential etiological and epidemiological traits with CD [19-21]. We have recently shown a positive association between antibiotic use in early childhood and subsequent CD [22]. However, there are few data whether antibiotic exposure in *pregnancy* influences the risk of CD in offspring.

## Methods

In this prospective population-based cohort study we used data from the ABIS cohort (All Babies in Southeast Sweden) in order to examine the association between antibiotic exposure in pregnancy and CD in the offspring.

### Study population

Between October 1997 and October 1999, all parents to babies born in southeast Sweden were invited to participate in the ABIS cohort. Of the 21,700 babies born during the study period, the parents of 17,055 children (78.6%) gave their informed consent to participate. In the maternity ward (at childbirth), parents to 16,285 children completed a questionnaire that included questions on antibiotics use in pregnancy, maternal education level, heath status and first-degree heredity for CD, type 1 diabetes mellitus and other autoimmune diseases.

**Figure 1:**
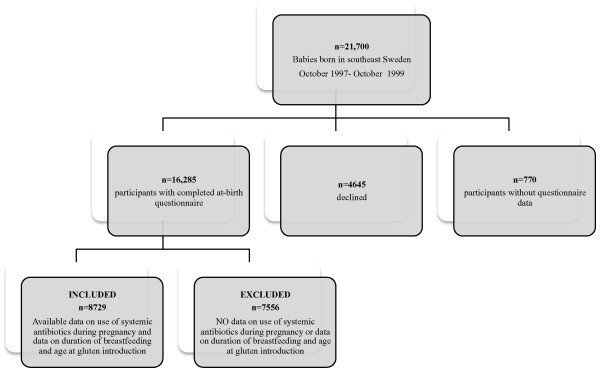
Flow chart study participants.

Parents were asked to complete a structured study diary during the child’s first year of life reporting infectious diseases and feeding practice, including duration of breastfeeding and age at gluten introduction. The diaries were completed prospectively at home and collected when the child was one year of age. In our main analysis we included 8729 individuals with data on use of systemic antibiotics during pregnancy and with complete diary data for duration of breastfeeding and time of gluten introduction (see flow chart, Figure 1).

The parents of the study participants were slightly more often born in Sweden and with a higher level of education, as compared with the source population of southeast Sweden [23]. Additional background data of the ABIS cohort have been described elsewhere [24,25].

### Coeliac disease (CD)

Data on CD were collected through contact with all paediatric departments (n = 8) in the ABIS study area. The majority of children with CD were identified through a study published in 2004 [24]. In 2007–2008, we again contacted the same paediatric departments and asked them to report additional ABIS children with a biopsy-verified CD (villous atrophy) diagnosed until December 1st 2006. In addition to a biopsy suggestive of CD (Marsh grade III), children with CD were required either to have CD-specific antibody markers or CD-consistent symptoms that resolved after introduction of gluten-free diet [24]. In the current study, date of CD diagnosis equals date of first positive small-intestinal biopsy. The ABIS population was not actively screened for CD and therefore the children with CD were investigated due to clinical manifestations of possible CD.

### Maternal antibiotic exposure in pregnancy

We collected questionnaire data on use of any systemic antibiotics during pregnancy. The mothers exposed to antibiotics were asked to specify the name on the type of antibiotics. Mothers who were uncertain of their antibiotic use in pregnancy (n = 27) or who had only used non-systemic antibiotics (n = 2) were excluded from the study.

### Statistical analyses

We used Cox regression to estimate Hazard Ratios (HRs) and 95% confidence intervals (CIs) for the risk of childhood CD according to maternal antibiotic exposure in pregnancy.

In our main analysis (Model A, n = 8729) we used diary data to adjust for duration of breastfeeding (0–2; 3–4; 5–6; 7–8; 9–10 and ≥11 months) and age at gluten introduction (0–2; 3–4; 5–6; 7–8; 9–10 and 11–12 months of age) as potential confounders for CD development [25-27].

In a second analysis (Model B, n = 8698) we also adjusted for maternal education level (≤12 vs. >12 years) and number of any parent-reported infectious disease in the child’s first year of life, categorized into the following months of age: 0–2; 3–4; 5–6; 7–8; 9–10 and 11–12 months of age. Education level has been associated with antibiotic use [28] and may influence the risk of CD diagnosis [29]. Also infectious load in early childhood may be associated with subsequent CD [30]. Follow-up began at child’s birth and ended at time of CD diagnosis or December 1st 2006 (end of follow-up). However, because CD only develop after gluten introduction, which often occurs during the first year of life, we also performed a Cox regression in which the time scale began at one year of age until CD diagnosis or December 1st 2006 (end of follow-up).

Mode of delivery and gestational age pertain a great impact on the infant gut colonization [10,31-33] and have been associated with an increased risk of CD in offspring [6,34]. In two subanalyses we therefore restricted our data to term deliveries and children born through vaginal delivery, respectively. We also performed stratified analyses by infant sex and heredity for CD or type 1 diabetes mellitus.

Statistical significance was defined as 95% CIs not including 1.0. We used SPSS version 20.0 (SPSS, Inc, Chicago, IL) for the statistical analyses.

### Post-hoc analyses

As opposed to short duration of breastfeeding, no breastfeeding at all can have a different effect on gut colonization. In a post-hoc analysis we therefore chose to adjust for duration of breastfeeding splitting the first time category (0–2 months) into: 0–6 days and 7 days-2 months (keeping 3–4 months; 5–6 months etc.). In a second post-hoc analysis we estimated the risk of CD in offspring according to antibiotic exposure in pregnancy excluding children with a first-degree relative with autoimmune disease. According to data from the at-birth questionnaire we defined heredity for an autoimmune disease as presence of at least one first-degree relative with any of the following conditions: Goitre with hypothyroidism or hyperthyroidism, Grave `s disease, pernicious anaemia, systemic lupus erythematosus, Addison’s disease, any diabetes mellitus, gestational diabetes, CD, inflammatory bowel disease or rheumatoid arthritis.

To increase the statistical power of the study, we performed a post-hoc analysis including all 14,942 ABIS children with available data on antibiotic exposure in pregnancy. In this post-hoc analysis we used multiple imputation to replace missing values for infant nutrition data. However, since the statistics software SPSS cannot handle survival analyses with imputed data (the command "selection variable: rule: imputation > =1" only runs in linear regression and logistic regression), we first examined the odds ratio (OR) for future CD in our original dataset adjusting for infant nutrition (according to the covariates in Model A) before using multiple imputation to adjust infant feeding.

In a final post-hoc analysis we adjusted for the children’s use of antibiotics during their first year of life. Data on antibiotic use was collected through a questionnaire when the child was one year old. The number of antibiotic courses used during the child’s first year of life was classified into the following categories: no use; 1–2; 3–5; ≥6 courses of antibiotics. Regrettably, we largely lacked data on type of antibiotic agent used by the children.

### Power calculation

At a significance level of 0.05 we had an 80% power to detect a relative risk of 2.45 for CD in offspring to mothers treated with antibiotics during pregnancy.

### Ethics

This study was as part of the ABIS study approved by the Research Ethics Committees of Linköping University (Li 287–96) and Lund University (Lu 83–97). Mothers gave their written informed consent after careful written as well as oral information and information via videotape.

## Results

Out of the 8729 children included in the study, 1836 (21%) had been exposed to antibiotics during pregnancy (unexposed: 6893; Table 1). Type of antibiotics was only listed in a minority of children (n = 235), with penicillin V being the most common type of antibiotic (n = 147). The children were followed up to December 1st 2006, corresponding to an average age of eight years. At end of follow-up, 46 out of 8729 children were diagnosed with CD, yielding a baseline CD prevalence of 0.5%. Girls made up half of the children, and the average maternal age at delivery was close to 30 years (Table 1).

**Table 1:** Descriptive characteristics of individuals according to antibiotic exposure in pregnancy

	**Antibiotics**	**No antibiotics**
**Total**^**A**^ (%)	1836 (21.0)	6893 (79.0)
**Infant sex**		
Girls, n (%)	904 (49.2)	3342 (48.5)
Boys, n (%)	932 (50.8)	3551 (51.5)
**Heredity**^**B**^		
Coeliac disease, n (%)	30 (1.6)	84 (1.2)
Type 1 diabetes mellitus, n (%)	56 (3.1)	155 (2.2)
**Maternal characteristics**		
Age at delivery; mean +/− SD (years)	30.3 +/− 4.4	29.7 +/− 4.5
Maternal university education^C^, n (%)	715 (39.1)	2272 (33.1)

^A^Individuals with complete data on breastfeeding and gluten introduction.

^B^First-degree relative with coeliac disease/type 1 diabetes mellitus.

^C^>12 years of education at time of delivery.

Twelve (0.7%) exposed and 34 (0.5%) unexposed children developed CD, corresponding to a HR of 1.33 (95% CI = 0.69-2.56). Risk estimates did not change more than marginally after adjustment for duration of breastfeeding and age at gluten introduction (Model A: adjusted HR = 1.32, 95% CI = 0.69-2.56) or when adding first-year childhood infections and maternal education level (Model B: adjusted HR = 1.28, 95% CI = 0.66-2.48). We found unchanged HR:s with follow-up time starting at time of gluten introduction (data not shown). The relative risk estimates for CD after antibiotic exposure during pregnancy were similar in boys and girls (Boys: adjusted HR = 1.28; 95% CI = 0.41-3.96; Girls: adjusted HR = 1.36; 95% CI = 0.60-3.05). Moreover, antibiotic exposure during pregnancy did not seem to favour an earlier appearance of CD (median age at CD diagnosis was some 2 years and 9 months among both children exposed and unexposed to antibiotics during pregnancy).

Some 316 children had a first-degree relative with either CD (n = 105) or type 1 diabetes mellitus (n = 202) or with both CD and type 1 diabetes (n = 9). Restricting our data to children without heredity for CD and type 1 diabetes did not change our risk estimates (adjusted HR = 1.22; 95% CI = 0.58-2.59). Neither did the risk estimates change appreciably when we restricted our data to children born full-term (n = 8270 [94.7%]) or with a vaginal delivery (n = 7647 [87,6%]) (Table 2).

**Table 2:** Risk of coeliac disease in offspring (follow-up from birth) according to antibiotic exposure in pregnancy

	**Antibiotics (%)**	**No Antibiotics (%)**	**Crude HR; 95% CI**	**Adjusted HR; 95% CI**^**A**^	**Adjusted HR; 95% CI**^**B**^
**All**	12/1836 (0.7)	34/6893 (0.5)	1.33; 0.69-2.56	1.32; 0.69-2.56	1.28; 0.66-2.48
**Sex**					
Boys	4/932 (0.4)	12/3551 (0.3)	1.27; 0.41-3.94	1.28; 0.41-3.96	1.37; 0.44-4.27
Girls	8/904 (0.9)	22/3342 (0.7)	1.35; 0.60-3.02	1.36; 0.60-3.05	1.21; 0.53-2.73
**Subgroups**					
No heredity^C^	9/1753 (0.5)	28/6660 (0.4)	1.22; 0.58-2.59	1.22; 0.58-2.59	1.16; 0.55-2.47
Term deliveries^D^	12/1739 (0.7)	32/6531 (0.5)	1.41; 0.73-2.74	1.41; 0.73-2.74	1.37; 0.70-2.66
Vaginal delivery	10/1584 (0.6)	30/6063 (0.5)	1.28; 0.63-2.61	1.28; 0.63-2.62	1.25; 0.61-2.56

^A^In Model A we adjusted for duration of breastfeeding and age at gluten introduction. Children with complete data on breastfeeding and gluten introduction were included in the analyses (n varied between 7647 and 8729).

^B^In Model B we adjusted for any parent-reported infection during the child’s first year of life, maternal education level as well as duration of breastfeeding and age at gluten introduction. Children with complete data on breastfeeding, gluten introduction as well as education level were included in the analyses (n varied between 7622 and 8698).

^C^Excluding individuals with a first-degree relative with celiac disease (n = 105), type 1 diabetes mellitus (n = 202) or both celiac disease and type 1 diabetes (n = 9).

^D^Full-term, ≥37 gestational weeks.

Hazard ratios (HR) estimated through Cox regression. Follow-up from birth.

Because CD only develops after gluten introduction (typically occurring within the first year of life), we also performed a number of pre-planned Cox regression analyses in which the time scale began at one year of age, thereby excluding six children diagnosed with CD before that age. Antibiotic exposure during pregnancy was not significantly associated with CD in offspring diagnosed after age one year (adjusted HR = 1.42; 95% CI = 0.71-2.83). The risk estimates for CD diagnosed after age one year stratified by sex and restricted to children with no heredity for CD and type 1 diabetes are presented in Table 3.

**Table 3:** Risk of coeliac disease in offspring (follow-up from one year of age) according to antibiotic exposure in pregnancy

	**Antibiotics (%)**	**No Antibiotics (%)**	**Crude HR; 95% CI**	**Adjusted HR; 95% CI**^**A**^	**Adjusted HR; 95% CI**^**B**^
**All**	11/1835 (0.6)	29/6888 (0.4)	1.43; 0.71-2.86	1.42; 0.71-2.83	1.37; 0.68-2.76
**Sex**					
Boys	4/932 (0.4)	8/3547 (0.2)	1.90; 0.57-6.32	1.91; 0.58-6.35	2.00; 0.60-6.66
Girls	7/903 (0.8)	21/3341 (0.6)	1.24; 0.53-2.91	1.24; 0.53-2.92	1.13; 0.48-2.67
**Subgroups**					
No heredity^C^	8/1752 (0.5)	24/6656 (0.4)	1.27; 0.57-2.82	1.26; 0.57-2.81	1.21;0.54-2.70
Term deliveries^D^	11/1738 (0.6)	27/6526 (0.4)	1.53; 0.76-3.09	1.52; 0.76-3.07	1.48; 0.73-2.99
Vaginal delivery	9/1583 (0.6)	25/6058 (0.4)	1.38; 0.64-2.96	1.37; 0.64-2.94	1.34; 0.62-2.88

^A^In Model A we adjusted for duration of breastfeeding and age at gluten introduction. Children with complete data on breastfeeding and gluten introduction were included in the analyses (n varied between 7641 and 8723).

^B^In Model B we adjusted for any parent-reported infection during the child’s first year of life, education level as well as duration of breastfeeding and age at gluten introduction. Children with complete data on breastfeeding, gluten introduction as well as education level were included in the analyses (n varied between 7616 and 8692).

^C^Excluding individuals with a first-degree relative with celiac disease (n = 105), type 1 diabetes mellitus (n = 202) or both celiac disease and type 1 diabetes (n = 9).

^D^Full-term, ≥37 gestational weeks.

Hazard ratios (HR) estimated through Cox regression. Follow-up from one year of age.

### Post-hoc analyses

In our main analysis we identified 64 children with less than 7 days of breastfeeding. In a post-hoc analysis adjusting for no breastfeeding (duration <7 days), age at gluten introduction, infectious load in the first year of life and maternal education level we found largely unchanged risk estimates (adjusted HR = 1.29; 95% CI = 0.66-2.49).

Finally, we excluded children with first-degree relatives with any autoimmune disease (remaining n = 7785 [89,2%]; HR = 1.40; 95% CI = 0.65-2.99).

To address the issue of low statistical power we performed a post-hoc analysis including all 14,942 children with available data on antibiotic exposure in pregnancy. We used multiple imputation to replace missing values for infant nutrition data. However, since the statistics software SPSS cannot handle survival analyses with imputed data, we first examined the OR for future CD in our original dataset adjusting for infant nutrition (maternal antibiotics: OR for offspring CD = 1.32; 95% CI = 0.68-2.56). Using multiple imputation when adjusting for infant feeding did not change the risk estimate more than marginally (OR = 1.10; 95% CI = 0.64-1.89).

In a post-hoc analysis, including 6773 children with complete diary data and available data on use of antibiotics during the first year of life, we found only marginally changed risk estimates for CD (adjusted HR = 1.65; 95% CI = 0.83-3.28). The association between CD and use of antibiotics during the child’s first year of life has been presented in Additional file 1, Table A1.

## Discussion

In this cohort study, we examined the association between maternal use of antibiotics in pregnancy and risk of CD in the offspring. The rationale for this study was the increasing amount of data indicating that the maternal intestinal microbiota in pregnancy influences the foetal and infant immune system development [35]. However, we found no statistically significant association between antibiotic exposure in pregnancy and CD in the offspring. Although this may be reassuring, our study does not rule out a modestly excess risk of CD in children to mothers who were treated with antibiotics during pregnancy.

A strength of this study is its prospective design, where data on antibiotic exposure in pregnancy were collected before CD diagnosis, eliminating the risk of recall bias. We also used prospectively collected data on infant feeding and infectious diseases during the child’s first year of life. Infections were measured through concurrent parent-reporting, and not through patient charts, because most Swedish infants with minor infections do not seek medical advice. Although, the infections were not recorded using standardized self-reporting criteria we do not suspect this potential risk of misclassification to be differential between children exposed/unexposed to antibiotics in pregnancy.

Similar to other birth-cohort studies [36,37], the current study suffers from attrition. Half of the population entering at birth completed the 1-year-diaries, and there was a slight underrepresentation of parents with foreign origin and low education [38]. However, this should not influence the risk estimates if the association between maternal antibiotic exposure and risk of offspring CD is independent of education status. We are not aware of any study indicating that education modifies the risk factors of CD, even if socioeconomic status per se may be linked to the risk of CD [39,40].

Of greater concern is the limited statistical power of this study and the correspondingly increased risk of type 2 error (i.e. to erroneously accept a false null hypothesis). Additionally, we have not been able to screen the ABIS cohort for CD, and our patients consist of children with clinically diagnosed CD. The baseline CD prevalence of 0.5% (46 out of 8729 children) is therefore considerably lower than the estimated 2% prevalence of *screening*-detected CD in Swedish children [41] (in our study corresponding to 175 children). However, false-negative CD in our control group (<2% [41]) should not affect our risk estimates. Further, we do not expect risk factors to differ between children with or without symptoms (meriting investigation for CD). Unfortunately we lack data on clinical phenotype and were unable to examine if intrauterine antibiotic exposure is linked to a certain type of CD.

Data on antibiotics during pregnancy were collected retrospectively (at childbirth) and we did not have data on the specific date of the antibiotic treatment or on intrapartum antibiotics. Speculatively, antibiotic treatment during the perinatal period may have a more profound impact on infant gut colonization, as compared with antibiotic treatment early in pregnancy [11]. Additionally, we largely lack data on type of antibiotic agent, its dosage, duration of treatment as well as the indication for treatment, which all influence the mother’s microbiota, and that of the offspring [42]. The lacking specificity regarding type of antibiotic exposure may also have contributed to an increased risk of a type 2 error. The type of antibiotic used by the mother should also be linked to the type of infections she experienced during pregnacy and her inflammatory status that *per se* may also influence fetal immune programming and risk of autoimmune disease in the offspring.

It is well-established that the postnatal intestinal microbiota influences the maturation of the intestinal immune system and that individuals with CD, or with an increased genetic risk of developing CD, have an imbalanced intestinal microbiota, which may potentially enhance the inflammatory response elicited by gluten [9,43-45]. Animal studies suggest that the intestinal dysbiosis associated with CD may, in the presence of gliadin, increase the permeability of the small-intestine [9] and enable epithelial translocation of gliadin that may trigger CD [9,46]. Although the current study lacks evidence for a *prenatal* association between antibiotics and CD, our results do not refute the hypothesis that the early intestinal microbiota affects CD development.

Postnatal microbial exposure is likely to play a greater role in immune maturation and thereby CD development as compared with prenatal microbial exposure [35]. Still, vast amount of data suggest that pregnancy is a critical time for immune development and epigenetic models of allergic outcomes suggest that foetal immune development may be influenced by epigenetic modifications from microbial products [35]. In addition, several epidemiological studies have shown that prenatal exposures to antibiotics are associated with the development of childhood asthma, a disease that shares potential etiological traits with CD [19-21].

## Conclusions

This study found no statistically significant association between maternal use of antibiotics during pregnancy and CD in the offspring. This lack of association may either be true or due to limited statistical power.

## Abbreviations

ABIS: All Babies in Southeast Sweden; CD: Celiac disease; CI: Confidence interval; HR: Hazard ratio; OR: Odds ratio.

## Competing interests

The authors declare that they have no competing interests.

## Authors’ contributions

ICMJE criteria for authorship read and met: KM, JFL, YS, JL. Agree with the manuscript’s results and conclusions: KM, JFL, YS, JL. Designed the experiments/the study: KM, JFL, JL. Collected data: JFL, JL. Analyzed the data: KM. Wrote the first draft of the paper: KM. Contributed to the writing of the paper: JFL, YS, JL. Contributed to design of study and interpretation of the data analyses: KM, JFL, YS, JL. Interpretation of data; approved the final version of the manuscript: KM, JFL, YS, JL. Responsible for data integrity: KM, JFL. Supervised the project including data analyses: JFL.Obtained funding: JFL, JL. All authors read and approved the final manuscript.

## Pre-publication history

The pre-publication history for this paper can be accessed here:

http://www.biomedcentral.com/1471-230X/14/75/prepub

## Supplementary Material

Additional file 1: Table A1 Risk of coeliac disease according to the child’s use of antibiotics in the first year of life. Hazard ratios (HR) estimated through Cox regression. Click here for file
