# Supplementary material for: Antibiotic exposure in pregnancy and risk of coeliac disease in offspring: a cohort study
Source: BMC Gastroenterol. 2014 Apr 14;14:75. doi: 10.1186/1471-230X-14-75 (PMC4021104; doi:10.1186/1471-230X-14-75)
Supplement: Additional file 1: Table A1 — Risk of coeliac disease according to the child’s use of antibiotics in the first year of life. Hazard ratios (HR) estimated through Cox regression. [file 1471-230X-14-75-S1.pdf]

Additional file.

Table A1. Risk of coeliac disease according to the child's use of antibiotics in the first year of life. Hazard ratios (HR) estimated through Cox regression.

Child's use of antibiotics in the first year of life	Crude HR; 95% CI
No use	Reference
1-2 courses	1.47; 0.75-2.88
3-5 courses	1.80; 0.54-6.05
≥6 courses	NE

NE, not estimated because of lack of events
